# Prokaryotic ubiquitin-like protein remains intrinsically disordered when covalently attached to proteasomal target proteins

**DOI:** 10.1186/s12900-017-0072-1

**Published:** 2017-02-01

**Authors:** Jonas Barandun, Fred F. Damberger, Cyrille L. Delley, Juerg Laederach, Frédéric H. T. Allain, Eilika Weber-Ban

**Affiliations:** 1 ETH Zurich, Institute of Molecular Biology & Biophysics, Zürich, CH-8093 Switzerland; 2 Present address: Laboratory of Protein and Nucleic Acid Chemistry, The Rockefeller University, New York, NY USA

**Keywords:** Pupylation, Prokaryotic ubiquitin-like protein Pup, *Mycobacterium tuberculosis*, NMR, Intrinsically disordered proteins

## Abstract

**Background:**

The post-translational modification pathway referred to as pupylation marks proteins for proteasomal degradation in *Mycobacterium tuberculosis* and other actinobacteria by covalently attaching the small protein Pup (prokaryotic ubiquitin-like protein) to target lysine residues. In contrast to the functionally analogous eukaryotic ubiquitin, Pup is intrinsically disordered in its free form. Its unfolded state allows Pup to adopt different structures upon interaction with different binding partners like the Pup ligase PafA and the proteasomal ATPase Mpa. While the disordered behavior of free Pup has been well characterized, it remained unknown whether Pup adopts a distinct structure when attached to a substrate.

**Results:**

Using a combination of NMR experiments and biochemical analysis we demonstrate that Pup remains unstructured when ligated to two well-established pupylation substrates targeted for proteasomal degradation in *Mycobacterium tuberculosis*, malonyl transacylase (FabD) and ketopantoyl hydroxylmethyltransferase (PanB). Isotopically labeled Pup was linked to FabD and PanB by in vitro pupylation to generate homogeneously pupylated substrates suitable for NMR analysis. The single target lysine of PanB was identified by a combination of mass spectroscopy and mutational analysis. Chemical shift comparison between Pup in its free form and ligated to substrate reveals intrinsic disorder of Pup in the conjugate.

**Conclusion:**

When linked to the proteasomal substrates FabD and PanB, Pup is unstructured and retains the ability to interact with its different binding partners. This suggests that it is not the conformation of Pup attached to these two substrates which determines their delivery to the proteasome, but the availability of the degradation complex and the depupylase.

**Electronic supplementary material:**

The online version of this article (doi:10.1186/s12900-017-0072-1) contains supplementary material, which is available to authorized users.

## Background

In close analogy to eukaryotic ubiquitination, during the process of bacterial pupylation proteins are covalently tagged on lysine side chains with a small protein modifier, the prokaryotic ubiquitin-like protein (Pup), targeting them for proteasomal degradation [1, 2]. The formation of an isopeptide bond between the side chain carboxylate of the C-terminal glutamate of Pup and a protein substrate lysine is catalyzed by the Pup ligase PafA [3–5]. In mycobacteria and many other actinobacteria Pup is encoded in a coupling-incompetent pro-form featuring a glutamine at the C-terminus. In these organisms, modification of a substrate requires the action of a deamidase termed Dop (deamidase of Pup), converting the C-terminal glutamine of Pup into a glutamate [5]. Interestingly, aside from its function as deamidase, the enzyme Dop counteracts the ligase PafA, catalyzing the specific cleavage of the isopeptide bond between Pup and the substrate [6, 7]. This is why the *dop* gene is always present in the pupylation gene locus, even in organisms that encode *pup* in its ligation-competent form featuring a glutamate at the C-terminus [6, 7].

In marked contrast to the stable β-grasp fold of ubiquitin [8], the 64 residue Pup was shown to be an intrinsically disordered protein in its free form with helical propensity detected in residues 50–58 [9–11]. Upon interaction with its binding partners however, Pup adopts different structures. When binding to the mycobacterial proteasomal ATPase Mpa (referred to as ARC in other actinobacteria), residues 20 to 51 of Pup form an elongated helix associating into a shared coiled-coil with the N-terminal domains of Mpa [11, 12]. Contrastingly, when Pup interacts with the ligase PafA, the C-terminal half of Pup forms two orthogonal helices (H1: 38–47 and H2: 51–58) which interact with an extended shallow groove on the surface of PafA [13].

The ability of free non-ligated Pup to adopt alternate structures in response to different interaction partners raises the question, whether Pup changes its conformation when covalently attached to a target protein. This is of particular interest, as the conformational state could have an influence on the fate of pupylated target proteins.

In mycobacteria*,* proteomic analysis identified more than 600 pupylated proteins [14–16]. However, only a few targets have been verified experimentally to be degraded via the Pup proteasome pathway. Amongst the best characterized pupylation and proteasome targets, as confirmed by several in vivo studies, are FabD (Rv2243; malonyl Co-A:acyl carrier protein transacylase) and PanB (Rv2225; ketopantoate hydroxymethyltransferase), enzymes required for the biosynthesis of fatty acids and polyketides. PanB catalyzes the first committed step of pantothenate biosynthesis and is thus involved in the biosynthesis of coenzyme A [17], while FabD transfers the malonyl moiety from coenzyme A to acyl-carrier protein, producing the activated C2-donor in the synthesis of fatty acids, malonyl-ACP [18]. The homeostasis of both enzymes is dependent on the Pup proteasome degradation pathway and both proteins are essential for pathogenesis of *Mycobacterium tuberculosis* (Mtb) [17–20].

Here, we characterize the conformation of Pup when linked to either of these well-characterized proteasomal substrates, FabD and PanB, and compare the results with the conformation of Pup linked to free lysine. In the course of generating homogeneously pupylated PanB for NMR measurements, the previously unknown target lysine was identified by mass spectrometry and mutational analysis. Our results demonstrate that Pup, conjugated to either of the two investigated proteasomal substrates, adopts an intrinsically disordered state similar to the one observed in the free, unbound form. Substrate-tethered Pup retains high flexibility, and therefore entropy, as evidenced by the detection of NMR signals for Pup’s C-terminal residues even when attached to the ~300 kDa PanB decamer. This contrasts with the distinct ordered states observed for the C-terminal half of Pup interacting with either the ligase PafA or the ATPase Mpa.

## Results

### Production of homogeneously pupylated substrates suitable for NMR

FabD and PanB are confirmed pupylation and proteasome targets making them ideal candidates for studying the conformational state of Pup within the Pup-substrate conjugate. Although both Mpa substrates are of similar molecular weight (30 kDa), they differ in their oligomeric state; FabD is a monomeric protein of 30.8 kDa while PanB is a homodecamer of 293 kDa.

A prerequisite for structural characterization by NMR is the production of a highly concentrated Pup-substrate conjugate sample modified homogeneously at a specific lysine residue. While in vivo and in vitro analysis of various substrates has shown that the ligase PafA does not randomly modify all accessible surface lysines but rather acts specifically on certain residues, the specificity is rarely absolute [5, 15, 21]. In vitro conditions geared toward producing preparative amounts of the covalent complex often produce polypupylated products [5, 22]. Moreover, even in the context of the cell some substrates show more than one modifiable lysine [14–16, 21]. This potential heterogeneity needed to be addressed before an isotopically labeled complex could be produced. FabD of Mtb contains eight lysines that are all located on the surface of the monomeric protein. Of those eight lysines three were previously reported to be Pup modification sites (K122, K173 and K181) [21]. Amongst those, K173 appeared to be the main pupylation target, since the half-life of the FabD-K173A variant is greatly increased in vivo [1].

**Fig. 1:**
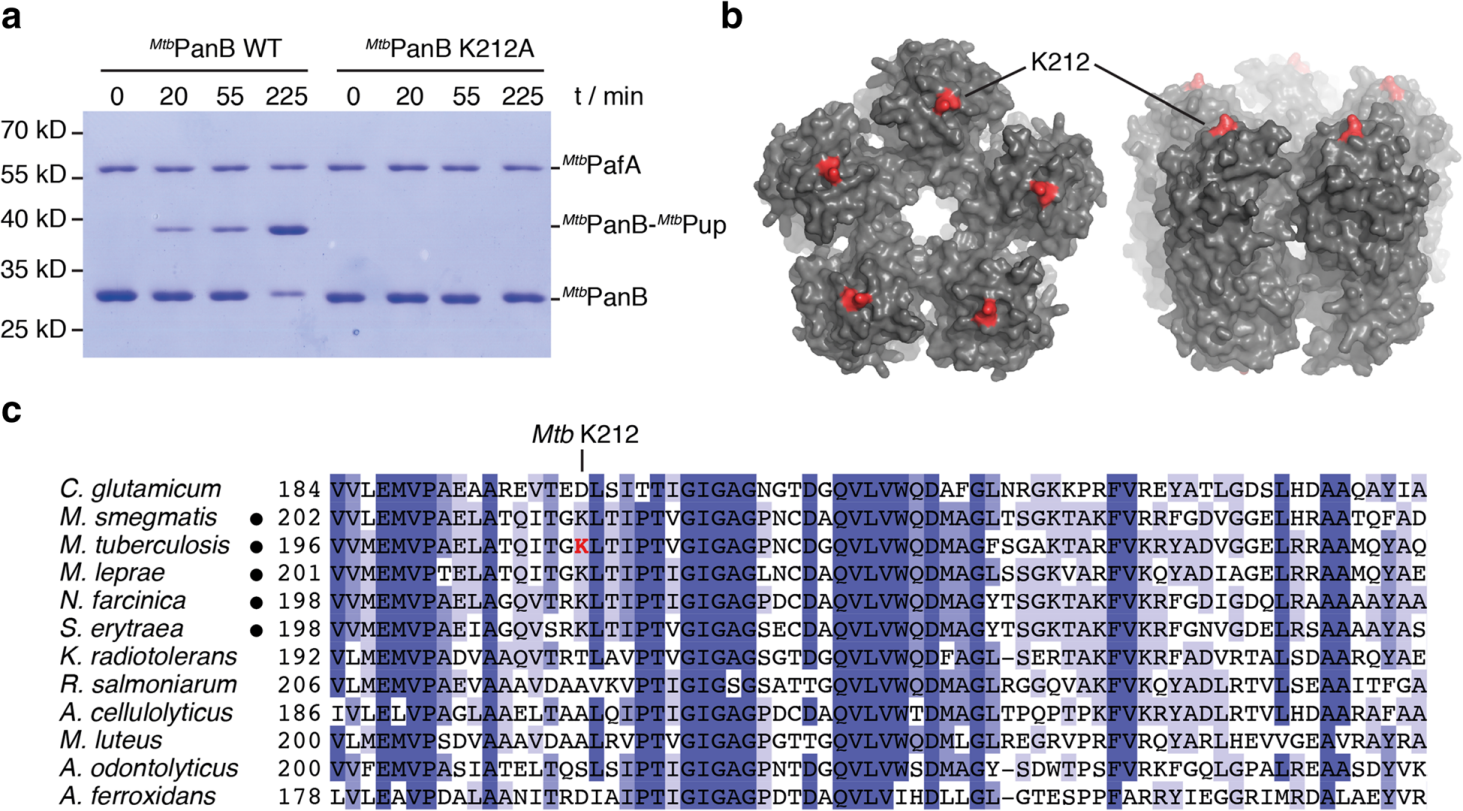
PanB from *Mycobacterium tuberculosis* is pupylated at a single lysine residue (K212). **a** Formation of a covalent ^*Mtb*^Pup ~ ^*Mtb*^PanB conjugate analysed by SDS–PAGE and Coomassie staining after incubation of 6 μM ^*Mtb*^PanB and 12 μM ^*Mtb*^Pup with 1 μM ^*Mtb*^PafA in the presence of 5 mM ATP. The variant K212A does not show formation of a conjugate band over the same time-course. **b** Top and sideview of the ^*Mtb*^PanB decamer (pdb code 1OY0) in surface representation (*grey*). The pupylated lysine K212 is *highlighted in red*. **c** Alignment of PanB orthologs from representative actinobacteria with only the portion around K212 shown. Lysine K212 of ^*Mtb*^PanB (shown in *red*) is conserved in all proteasome-harboring actinobacteria (indicated in the alignment by a *black dot*). The degree of conservation is expressed as different *shades of blue* with low conservation *light blue* and high conservation *dark blue*

**Fig. 2:**
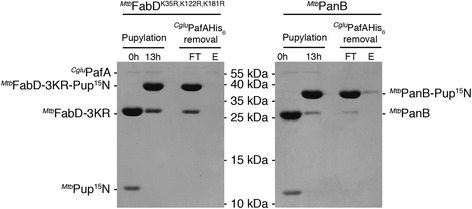
Preparative pupylation of ^*Mtb*^FabD-3KR (K35R, K122R and K181R) and ^*Mtb*^PanB with ^15^N-labeled ^*Mtb*^Pup. Pupylation of 40 μM ^*Mtb*^PanB or 40 μM ^*Mtb*^FabD-3KR with 35 μM ^15^N-^*Mtb*^Pup in the presence of 10 mM ATP and 2.5 μM ^*Cglu*^PafA-His_6_ in 2500 μl total reaction volume and subsequent removal of the Pup ligase using a Ni-NTA spin-column (FT: flowthrough, E: elution with 250 mM Imidazole)

In contrast to ^*Mtb*^FabD, the pupylation target lysine of ^*Mtb*^PanB has not been identified. PanB was one of the first proteasomal substrates to be described, it accumulates in pupylation- and proteasome-deficient mutant strains and it is a widely used model substrate for in vitro studies of the Pup proteasome system [5, 22, 23]. One ^*Mtb*^PanB monomer contains eight lysines with six located on the solvent-accessible surface of the decamer (K20, K30, K35, K212, K243, K249). In a pupylation time course of PanB, a single band corresponding to pupylated PanB was observed by Coomassie-stained SDS-PAGE (Fig. 1a). This indicates that PanB is either exclusively modified at one specific lysine or is singly modified at multiple lysine residues which are close enough in space, such that modification of the first prevents pupylation of the second lysine in the same protomer. To identify the modified lysine, we analyzed the protein band corresponding in size to the covalent adduct of PanB (29.3 kDa) and Pup (7 kDa) by tandem mass spectrometry. Using this strategy, a sequence coverage of 45% was obtained, but no peptide containing a modified lysine could be identified (Additional file 1: Figure S1a). However, the sharp single Pup ~ PanB gel band observed upon pupylation of PanB suggests that only one specific lysine is modified. Hence, the unmodified lysines occurring in the observed peptides were excluded as targets. Furthermore, those lysines that remained accessible for tryptic digest after completing the pupylation reaction are unlikely targets, because modified lysines are not cleaved by trypsin. This left two lysines (K212 or K243) as potential targets. Mutating the candidate lysine K212 (Fig. 1b and c) revealed it as the predominantly pupylated target lysine, since the PanB-K212A variant no longer serves as a pupylation substrate over the same time course during which wild type PanB was pupylated almost to completion (Fig. 1a). This suggests that a homogeneous Pup ~ K212-PanB product is formed in the reaction catalyzed by the ligase PafA. The identified lysine K212 is located in a long, otherwise lysine- and arginine-free, peptide (G175 to A242), which explains the difficulty of observing it directly in the mass spectrometric analysis (lysines modified with Pup are not cleaved by trypsin). For NMR analysis, preparative amounts of Pup ~ K212-PanB were produced using ^15^N-labeled Pup (Fig. 2).

To create a homogeneous sample of FabD modified at a single lysine, we mutated the two reported secondary pupylation lysines (K122 and K181) [21] to arginines. Surprisingly, even after introducing these two mutations more than one Pup ~ FabD band was observed on SDS-PAGE, suggesting that a fourth lysine was modified by PafA (Additional file 1: Figure S1b). Analysis of the corresponding bands by MS/MS identified K35 as the fourth modifiable lysine (Additional file 1: Figure S1b and c). A triple lysine-to-arginine variant was therefore generated, which removed not only the two previously reported secondary sites but also lysine K35 (FabD-3KR; containing the mutations K35R, K122R and K181R). A homogeneously pupylated sample of ^15^N-Pup ~ FabD-3KR was prepared for NMR measurements by in vitro pupylation (Fig. 2).

### Narrow dispersion of ^1^H and ^15^N chemical shifts indicates substrate-linked ^*Mtb*^Pup is disordered

It has been shown previously that free Pup in solution is mainly disordered with a weakly populated short helical segment at the C-terminal end ranging from residue 50 to 58 [9–11]. The [^15^N,^1^H]-HSQC spectrum of free Pup exhibits a very narrow chemical shift dispersion for the ^1^H-^15^N signals, which is a characteristic feature of unfolded proteins [9–11]. When interacting with PafA, the region showing some helical propensity in free Pup (50–58) adopts a helical conformation, corresponding to the second of the two orthogonal helices observed in the co-crystal structure of Pup with the ligase PafA [13]. This same region is, however, disordered when bound to the coiled-coil domain of Mpa, where the middle part of Pup forms a single long helix (21 to 50) [12].

In order to assess which conformational state Pup adopts when covalently attached to a substrate, we measured [^15^N,^1^H]-HSQC spectra of the recombinantly generated Pup-substrate conjugates ^15^N-Pup ~ FabD-3KR and ^15^N-Pup ~ PanB. In addition, we performed triple resonance experiments to obtain ^13^Cα shifts for ^13^C^15^N-Pup ~ FabD-3KR. Measurements were carried out under the same conditions used for Pup coupled to the free amino acid lysine (Pup ~ lysine), which served as a reference.

**Fig. 3:**
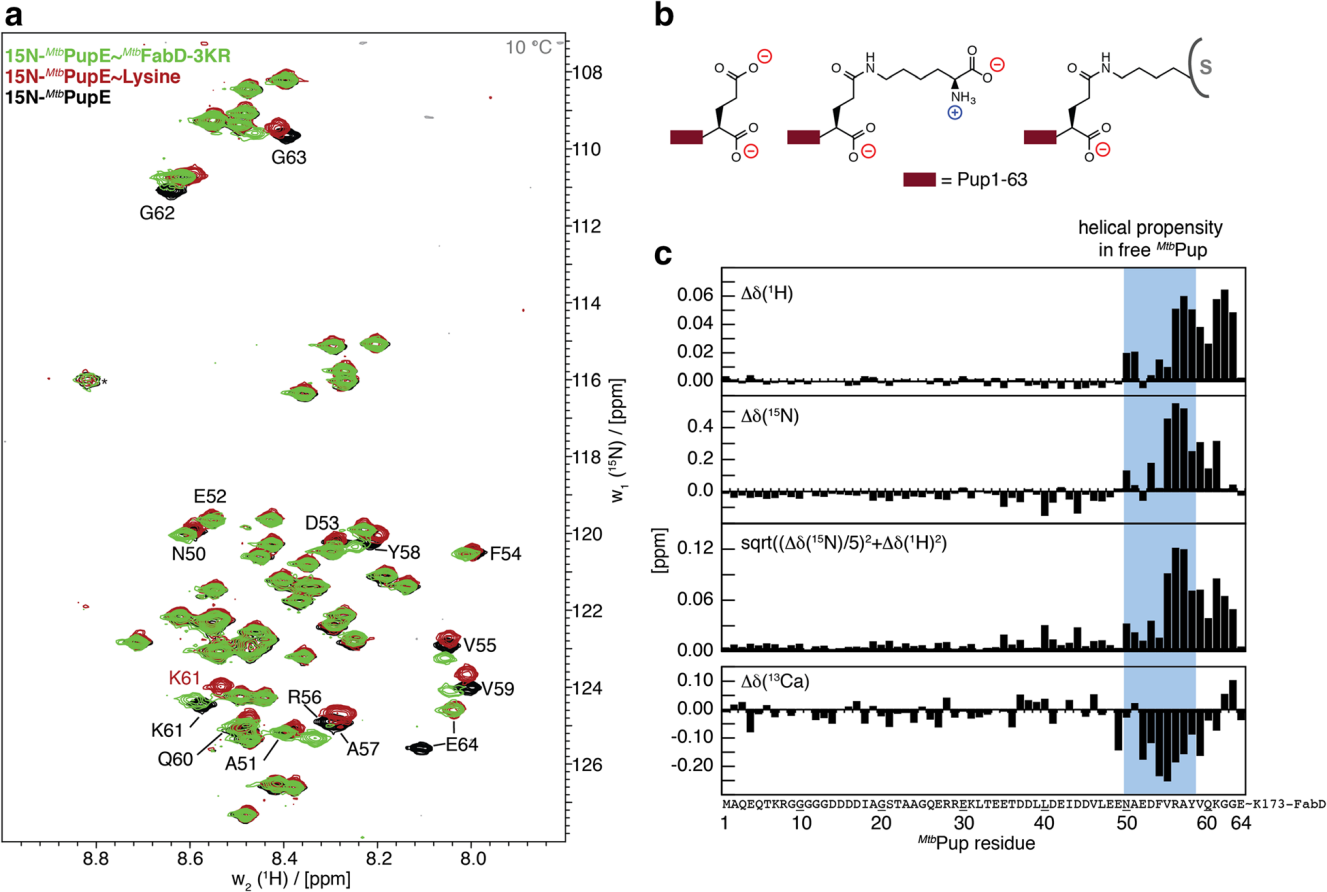
Chemical shifts of ^15^N-labeled ^*Mtb*^Pup conjugated to ^*Mtb*^FabD-3KR compared with those of ^15^N-labeled ^*Mtb*^Pup conjugated to lysine or with free ^15^N-labeled ^*Mtb*^Pup. **a** Superposition of [^15^N,^1^H]-HSQC correlation spectra of ^15^N-^*Mtb*^Pup (*black*) with ^15^N-^*Mtb*^Pup ~ lysine (*red*) and ^15^N-^*Mtb*^Pup ~ ^*Mtb*^FabD-3KR (*green*), all measured at 10 °C. **b** Schematic representation of the C-terminal end of ^*Mtb*^Pup present in the three NMR samples highlights the different charge states. S stands for substrate (^*Mtb*^FabD or ^*Mtb*^PanB). **c** Histograms of the absolute chemical shift of ^*Mtb*^Pup ~ ^*Mtb*^FabD-3KR minus ^*Mtb*^Pup ~ lysine and the traditional chemical shift mapping based on the weighted sum of ^1^H and ^15^N shift changes. The region with helical propensity in free ^*Mtb*^Pup is indicated in *blue*

In the [^15^N,^1^H]-HSQC spectrum of Pup coupled to FabD-3KR (Fig. 3a), which is the smaller of the two investigated substrates, all previously assigned residues of free Pup [9–11] and Pup ~ lysine [3] could be observed. The first 50 residues show negligible chemical shift changes indicating that there are no significant conformational differences compared to Pup ~ lysine in this region (Fig. 3c). The ^1^H^N^ and ^15^N resonances of residues 50 to 58 exhibit increased chemical shifts in the conjugated complex and the ^13^Cα shifts are slightly reduced in this region compared to Pup-lysine (Fig. 3c, region with blue background). Both trends suggest a modest decrease in helical propensity [24–26]. Secondary shifts show a similar trend when comparing the FabD-3KR-conjugated Pup to Pup-lysine (Additional file 2: Figure S2).

Overall, the very small chemical shift changes relative to Pup ~ lysine indicate dynamically disordered behavior of FabD-3KR-conjugated Pup with a decreased propensity for helical conformation in the PafA interaction region of Pup.

**Fig. 4:**
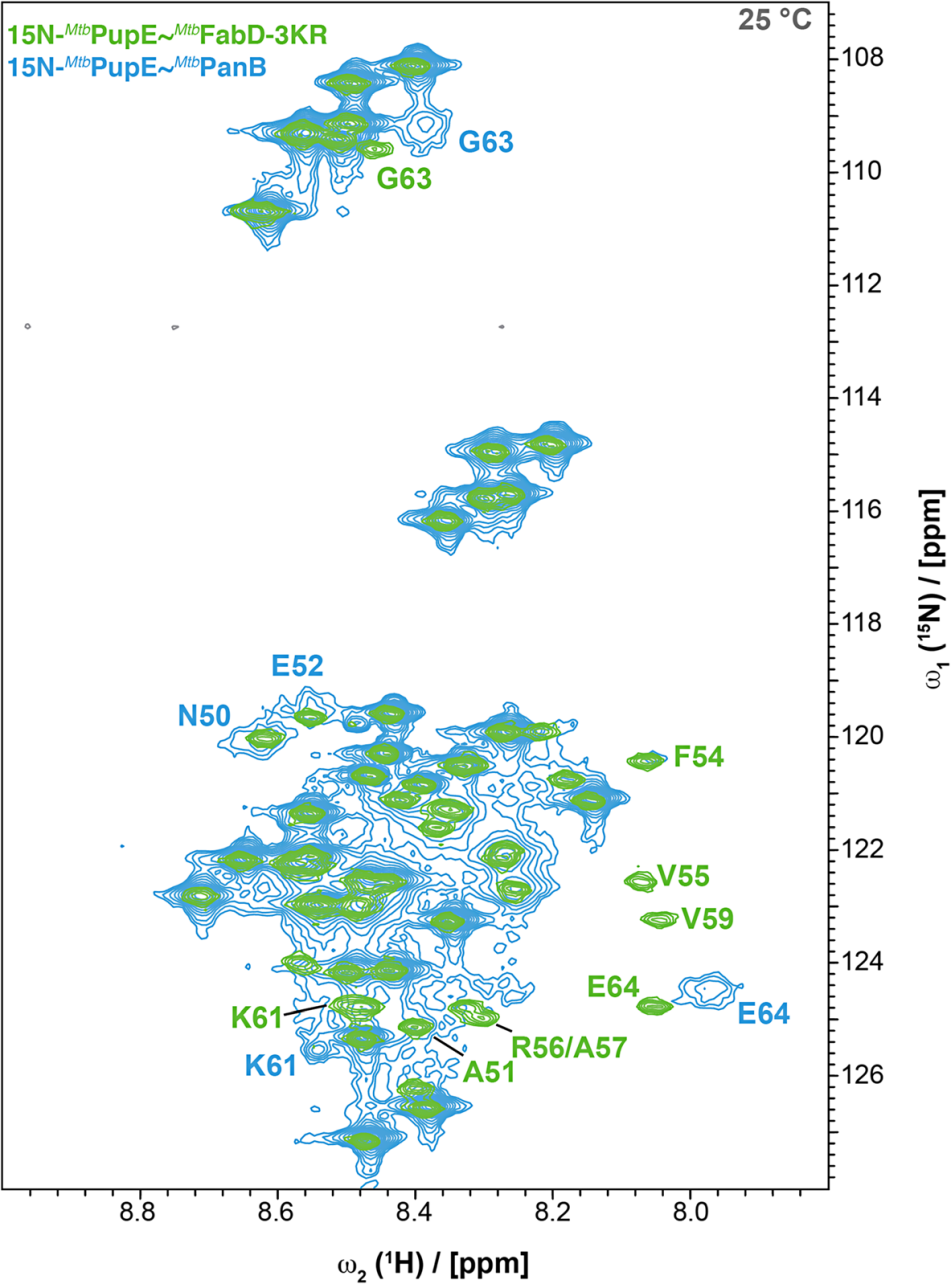
Narrow dispersion of the chemical shifts in the [^15^N,^1^H]-HSQC spectra of ^15^N-labeled ^*Mtb*^Pup conjugated to ^*Mtb*^PanB or ^*Mtb*^FabD-3KR indicate disordered state of ^*Mtb*^Pup. Superposition of ^15^N-^*Mtb*^Pup ~ ^*Mtb*^PanB in *blue* with ^15^N-^*Mtb*^Pup ~ ^*Mtb*^FabD in *green*, both measured at a temperature of 25 °C

While all residues of Pup when attached to the monomeric substrate FabD could be detected in the [^15^N,^1^H]-HSQC spectra, for Pup coupled to the PanB decamer, approximately the last 26 residues were not visible under the same conditions (10 °C, Additional file 3: Figure S3). This may be due to the almost one order of magnitude larger size and thus about 10-fold slower rotational relaxation time of this oligomeric substrate (293 kDa of the PanB decamer versus 30.8 kDa for the FabD monomer). To increase the tumbling rate, the experiment was therefore conducted at elevated temperature (Fig. 4). At 25 °C, with the exception of a few residues near the C-terminus (such as F54, V55 and V59), most of the 26 C-terminal residues, surprisingly even including the C-terminal glutamate (E64), could be observed in an overnight [^1^H^15^N]-HSQC spectrum (Fig. 4). The significant broadening observed for residues 50–58 could be caused by the helical propensity in this region: when transiently in the helical state, the hydrophobic patch formed by F54, V55 and V59 could form contacts with PanB causing the residues to move on average with a slower time-scale, or, alternatively, changes in the stability of the transient helical state may lead to exchange broadening. As was observed in the case of Pup ligated to FabD, the [^15^N,^1^H]-HSQC spectrum of Pup ligated to PanB also exhibits a narrow shift dispersion indicative of an intrinsically disordered protein.

### Pup covalently ligated to a target protein remains accessible for interaction partners

If the conformational state of Pup changed upon conjugation to a substrate, this would be likely to change the ability to interact with its binding partners. To assess this, we measured the affinity of Pup ~ FabD and Pup ~ PanB to PafA (product binding). This experiment was performed rather than binding to the ATPase Mpa or to Dop, because the co-crystal structure of Pup with PafA [13] is available and shows the Pup-binding groove of PafA is fully accessible and that the active site β-sheet cradle features a shallow and open arrangement, so that only minor steric interference, if any, from the substrate protein portion itself is expected. Thus, product binding (pupylated substrate) can be compared to substrate binding (Pup) in the absence of enzymatic turnover and in absence of steric hindrance from the substrate protein portion. We produced both FabD and PanB samples homogeneously pupylated with a fluorescein-labeled Pup-Q30C variant from *Corynebacterium glutamicum (Cglu)*. This Pup variant has previously been used to determine the affinity of ^*Cglu*^Pup for ^*Cglu*^PafA using fluorescence anisotropy [13]. ^*Mtb*^PafA could not be used for the binding titrations due to its much lower solubility and therefore ^*Cglu*^PafA was used instead. As reported earlier, free ^*Cglu*^Pup binds to the ligase ^*Cglu*^PafA with a dissociation constant in the low micromolar range based on isothermal calorimetry and fluorescence anisotropy measurements [13, 27].

**Fig. 5:**
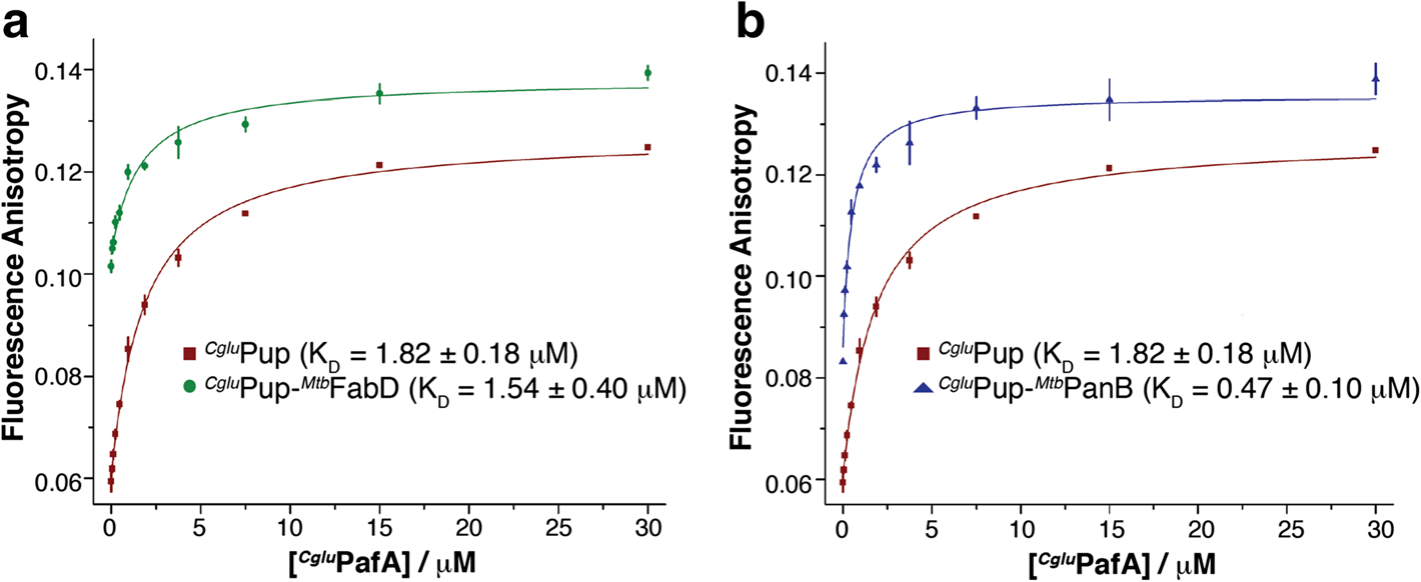
^*Cglu*^Pup covalently attached to a substrate remains accessible to ^*Cglu*^PafA and binds to it with similar affinities as free ^*Cglu*^Pup. ^*Cglu*^PafA: ^*Cglu*^Pup ~ substrate affinity measurements using fluorescence anisotropy with a fluorescein-labeled ^*Cglu*^Pup-Q30C variant. **a** Affinity of ^*Cglu*^PafA for ^*Cglu*^Pup ~ ^*Mtb*^FabD-3KR (*green*) or **b** ^*Cglu*^Pup ~ ^*Mtb*^PanB (*blue*) lies in a similar range as the affinity for free ^*Cglu*^Pup-Q30C (*red*, a and b)

While the previously performed fluorescence anisotropy affinity measurement was conducted on a PTI fluorimeter [13], here we used a Synergy BioTek plate reader and confirmed the low micromolar affinity of PafA for Pup (Fig. 5a and b; K_D_ = 1.82 ± 0.18 μM). The dissociation constant for binding of PafA to Pup that is covalently attached to substrate determined with this method falls into the same approximate range, with Pup ~ FabD-3KR exhibiting a K_D_ of 1.54 ± 0.40 μM (Fig. 5a) and Pup ~ PanB a K_D_ of 0.47 ± 0.10 μM (Fig. 5b). The slightly lower value for PafA binding to Pup ~ PanB compared to Pup could be caused by an avidity effect of multiple Pup proteins in close proximity ligated to the decameric PanB (Fig. 1b). Furthermore, unspecific low affinity interactions of PanB with PafA might make small contributions to the overall affinity. The measured dissociation constants demonstrate that the Pup binding site on PafA is not sterically inaccessible due to attachment of Pup to either substrate. More importantly, the result supports the notion that Pup linked to substrate is able to adopt different conformations, thereby remaining accessible for multiple interaction partners with different Pup-interaction surfaces. This is consistent with the NMR data showing that Pup linked to these substrates remains mainly disordered.

The crystal structure of Dop was determined in the absence of Pup or pupylated substrate, but according to homology modeling using the structure of PafA, the binding region of the last few residues of Pup is narrower, and NMR footprint data upon titration with Dop also reveal, that the four C-terminal residues of Pup are more constrained [27]. In agreement with this, the substrate portion was shown to cause steric hindrance in the binding of a pupylated substrate [7]. Nevertheless, Pup remains accessible for binding to the Pup-binding groove as demonstrated by affinity measurements using thermophoresis. We titrated randomly fluorescein-labeled Dop with either Pup or Pup ~ FabD, measuring dissociation constants in the medium to high nanomolar range (14.6 ± 3.3 nM for Pup and 157.4 ± 49.8 nM for Pup ~ FabD) (Additional file 4: Figure S4).

## Discussion

**Fig. 6:**
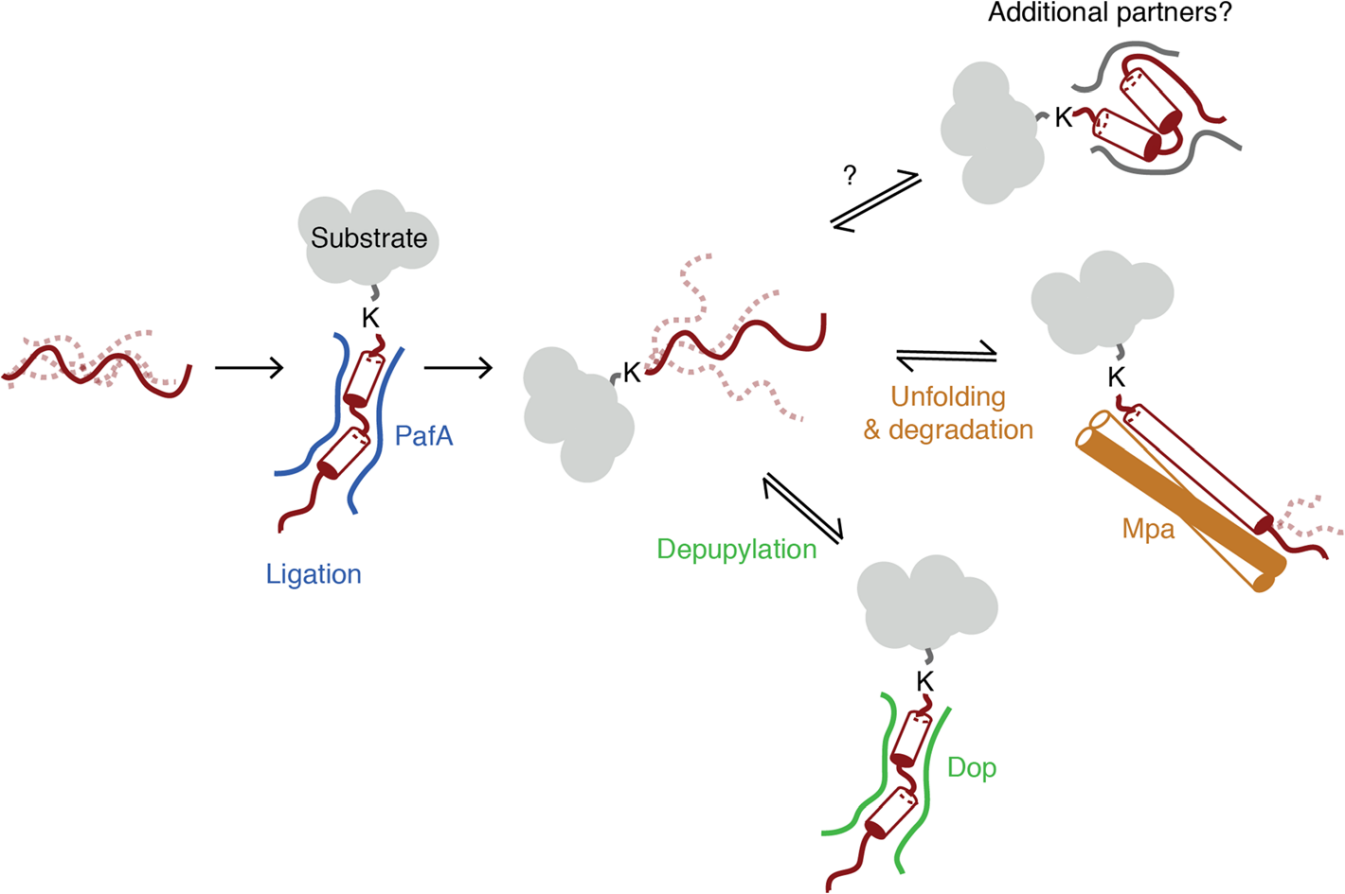
The disordered nature of Pup covalently ligated to a substrate allows for interaction with different Pup binding partners. Schematic representation of the disordered behavior of Pup (*red*) in its free form and when attached to a substrate (*grey*). The intrinsically disordered nature of Pup in its free as well as substrate-ligated form allows interaction with multiple binding partners such as the ligase PafA (*blue*), the deamidase/depupylase Dop (*green*), the mycobacterial proteasomal ATPase Mpa (*orange*) or additional unknown potential interaction partners

In eukaryotic cells, ubiquitination plays an essential role for homeostasis by marking proteins for proteasomal degradation [28]. While a subset of bacteria also harbors genes for the 20S proteasome, the molecular basis of tagging proteins for degradation is considerably different from ubiquitination [2]. Eukaryotic ubiquitination involves the attachment of the small globular protein ubiquitin to substrate lysines. In contrast, Pup is an intrinsically disordered protein that adopts a well-defined structure only when interacting with its binding partners such as the ligase PafA [13], the proteasomal ATPase Mpa [12] and based on the high degree of sequence and structural homology also the depupylase Dop [27] (Fig. 6).

However, whereas the conformation of Pup in its free form and while bound to the pupylation and degradation machinery has been described in a series of structural studies [9–13], the conformational state of Pup when covalently attached to a substrate protein remained unknown. The conformation of Pup in this context is of functional importance, as it could change the preference for one binding partner over another, which would in turn have an effect on the fate of the target protein modified with Pup. Alternatively, in adopting a different conformation at the surface of a substrate, Pup could make the Pup-binding surface required for interaction with Mpa or Dop unavailable, thus acting in an inhibitory mode.

NMR is an ideal method to address the conformational state of substrate-bound Pup, since it reports on the entire ensemble of coexisting conformations and also provides a measure of its flexibility and dynamics. Using this method, we show that Pup remains predominantly disordered when covalently attached to the two well-characterized proteasomal degradation substrates PanB and FabD. The narrow range of dispersion for the ^1^H^N^ and ^15^N chemical shifts, which is similar to that observed for free Pup attached to a single lysine, suggests that Pup tethered to a protein target is present as an ensemble of unfolded conformations rather than presenting one or a few folded states. In fact, the moderate helical propensity present in the C-terminal region of free Pup (residues 50–58) appears to be reduced when Pup is linked to FabD as evidenced by the increased chemical shifts in the ^1^H^N^ and ^15^N resonances and the decreased ^13^Cα shifts. FabD- or PanB-linked Pup thus remains conformationally available and fully accessible to different interaction partners. To further confirm this finding biochemically, we compared the affinity of Pup for PafA in its free and substrate-linked state to gauge the availability of binding-competent conformations of substrate-linked Pup. The fact that the affinity constant does not change significantly when Pup is ligated to FabD or PanB further supports that substrate-linked Pup has a conformational state similar to free Pup. Even in the case of the depupylase Dop, where steric hindrance due to the substrate protein portion of pupylated proteins has been demonstrated [7, 29], Pup tethered to Pup ~ FabD remains accessible for the binding groove on Dop as evidenced by the still rather tight binding constant of 157.4 nM.

Special features at the surface of a substrate protein, such as a long, accessible groove with residues complementing side chains featured on one face of a transiently populated helical stretch in the Pup C-terminal half could potentially trap certain conformational states of Pup. Such an interaction would then be similar in nature to the interaction between Pup and a true Pup-binding domain like the Pup-binding groove on PafA or the coiled-coil domain on Mpa. However, this is anticipated to be the exception rather than the rule, since it is difficult to imagine due to the diverse nature of the pupylome how each of these proteins could maintain a specific Pup binding site on their surface near the modified lysine(s).

Our findings support the hypothesis that the fate of the two verified degradation substrates, FabD and PanB, is not determined by a specific conformational state of Pup on the substrate but rather by the relative concentrations and availability of the binding partners of Pup. For example, increased levels of Mpa would favor degradation while increased levels of Dop would rescue pupylated substrates from destruction.

However, this should not be understood to mean that pupylated substrates behave like free Pup in their interaction with the pupylation enzymes and the proteasome. We have shown previously that the substrate protein portion can either add unspecific favorable interactions, as is the case for degradation by the Mpa-proteasome complex [22], or can introduce an element of sterical hindrance as we and others have shown for the depupylation reaction catalyzed by Dop [7, 29].

## Conclusions

Pupylation is an important post-translational modification occurring in actinobacteria and is capable of targeting proteins for proteasomal degradation. Despite the functional analogy to ubiquitination, the involved enzymes, their chemistry and the modifier protein Pup itself differ considerably from their eukaryotic functional counterparts [2].

Eukaryotic ubiquitin, with its distinct three-dimensional conformation [8], has been demonstrated to use a multitude of interaction modes with different binding partners (ubiquitin binding domains, UBDs), since the UBDs can make use of different surfaces of the ubiquitin fold [30]. The formation of poly-ubiquitin chains of different linkage further augments this repertoire of available surfaces. Conversely, as we show here, the interactions mediated by prokaryotic ubiquitin-like protein Pup when attached to a substrate protein are of an inherently different nature. Pup remains disordered in the covalently attached state and, as a result, remains competent for binding to different interactors requiring the adoption of different Pup folds, such as the proteasomal ATPase Mpa that unfolds the pupylated protein for degradation or the depupylase Dop that rescues the tagged protein by removal of Pup.

When Pup remains conformationally “naïve”, as shown here for two established proteasomal substrates, it can be envisioned that the fate of the pupylated target (degradation or rescue by depupylation) is determined by the relative concentrations of the various interaction partners of Pup. However, several factors could potentially influence degradation or depupylation: these include sterical effects or interactions with the substrate portion of the pupylated protein, so far unidentified additional regulatory protein factors or additional post-translational modifications such as phosphorylation.

## Methods

### Cloning

All primers (P1 to P14) used in this study are listed in Table 1.

**Table 1:** Primers used in this study

Constructs/Variants	Primer	Nr	Sequence of PCR primer (5'-3', restriction sites: *italic*, mutations/insertions: underlined)
His_6_-TEV-PanB	fwd	P1	ATTAGTTAT***CTTAAG***ATGTCGGAGCAGACTATCTAT
His_6_-TEV-PanB	rev	P2	AATTTATAT***CCTAGG***TTAGAAACTGTGTTCGTCAGC
PanB-K212A	fwd	P3	CCCAGATCACCGGC**GC**GCTTACCATTCCGACGG
PanB-K212A	rev	P4	TGGCCAACTCGGCGGGCACCATCTCCATCACGAC
His_6_-TEV-FabD	fwd	P5	GAGATATA***CATATG***CACCATCACCATCACCATGGCGAGAATCTTTATTTTCAGGGCATGATTGCGTTGCTGGCACCC
His_6_-TEV-FabD	rev	P6	ATATTAT***CCTAGG***TTATAGGTTTGCCAGCTCGTCCAGGT
His_6_-TEV-GG-FabD	fwd	P7	ATGATTGCGTTGCTGGCACCCGGA
His_6_-TEV-GG-FabD	rev	P8	**ACCGCC**GCCCTGAAAATAAAGATTCTCGCC
FabD-K35R	fwd	P9	GATCTTGCCCGGCTGGGCACCACC
FabD-K35R	rev	P10	TAGATCAGCGGC**ACG**CGACCACGC
FabD-K122R	fwd	P11	CGCGGCGCCGAGATGGCC**CGC**GCCTGCGCCACCGAGCCG
FabD-K122R	rev	P12	CGGCTCGGTGGCGCAGGC**GCG**GGCCATCTCGGCGCCGCG
FabD-K181R	fwd	P13	GACCCGCCGGCC**CGC**GCGCGGGTGCGT
FabD-K181R	rev	P14	TTCGGCGAGCTTCTCCAACGCGGTCAG

His_6_-TEV-^*Mtb*^PanB: The ^*Mtb*^ *panB* gene was amplified by PCR using primers P1 & P2. The resulting PCR fragment was then cloned into a previously used modified pET24 vector [13] using restriction enzymes *AflII* and *AvrII*. The final construct contains a N-terminal hexahistidine tag followed by a TEV cleavage site.

^*Mtb*^PanB-K212A: The construct used in [5] served as template for Quikchange site-directed mutagenesis using primers P3 & P4.

His_6_-TEV-GG-^*Mtb*^FabD & variants: The ^*Mtb*^ *fabD* gene was obtained by PCR using a previously cloned construct as template [5] by using primers P5 and P6. The PCR product was introduced into a modified pET24 vector [13] using the restriction sites *NdeI* and *AvrII*. Two additional glycines were introduced between the N-terminal TEV cleavage site and the protein sequence using primers P7 & P8. The mutation K122R (P11/P12) was introduced using the Stratagene Quikchange protocol while K181R (P13/P14), K35R (P9/P10) and the two additional glycines (P7/P8) were introduced using the Phusion site-directed mutagenesis protocol.

### Protein expression

The Pup ligase ^*Cglu*^PafA-His_6_ was purified as described in [27]. Briefly, ^*Cglu*^PafA was expressed from an IPTG-inducible pET24(+) plasmid in *E. coli* Rosetta cells for 16 h at 23 °C. After clearing the lysate by centrifugation for 60 min at 20’000 rpm, 4 °C, the ^*Cglu*^PafA was purified by nickel affinity chromatography using a 5 ml HisTrap HP column (GE Healthcare). Size exclusion chromatography was carried out on a Superose 6 pg gel filtration column equilibrated and run in 50 mM Tris, pH 7.5 (4 °C), 150 mM NaCl, 1 mM DTT, 1 mM EDTA and 10% glycerol.

The two N-terminally tagged substrates (His_6_-TEV-^*Mtb*^PanB, His_6_-TEV-^*Mtb*^FabD) including all their variants were expressed from IPTG-inducible plasmids in *E. coli* Rosetta cells for 16 h at 23 °C. The cells were harvested and the pellets were resuspended in 50 mM Tris, pH 7.5 (4 °C), 150 mM NaCl, supplemented with DNAse, Phenylmethylsulfonyl fluoride (PMSF) and Inhibitor Cocktail (Roche) and subsequently lysed by passing the solution four times through an Emulsiflex High Pressure Homogenizer. The solution was cleared by centrifugation at 20’000 rpm for 60 min at 4 °C and the protein was purified by nickel affinity chromatography using a 5 ml HisTrap HP column (GE Healthcare). Fractions containing the expressed protein were pooled, supplemented with TEV protease and subsequently dialyzed overnight at 4 °C against 50 mM Tris, pH 7.5 (4 °C), 150 mM NaCl using a dialysis membrane with a 14 kDa molecular weight cutoff. To remove uncleaved protein, the cleaved hexahistidine tag and the TEV protease, the protein solution was again passed over the 5 ml HisTrap HP column and further purified using a Superose 6 pg in buffer containing 50 mM Tris, pH 7.5 (4 °C), 150 mM NaCl, 1 mM DTT, 1 mM EDTA and 10% glycerol.

Isotopically labeled ^*Mtb*^Pup (^15^N or ^13^C^15^N) was expressed as described previously [3] with a few minor changes: *E. coli* Rosetta transformed with an IPTG-inducible plasmid carrying His_6_-Thioredoxin-TEV-^*Mtb*^PupGGE were grown in M9 minimal medium supplemented with ^13^C (99%) glucose as carbon source and/or ^15^N (98%) ammonium chloride as nitrogen source. Cells were induced with 1 mM IPTG at an OD_600_ of 0.7 and grown for 12 h at 37 °C. The white pellet (6.9 g cells for ^15^N-^*Mtb*^Pup or 7.3 g cells for ^13^C^15^N- ^*Mtb*^Pup) was resuspended in 35 ml lysis and wash buffer (l&w buffer: 100 mM Phosphate Buffer pH 7.6, 300 mM NaCl) supplemented with DNAse, PMSF and Roche Inhibitor Cocktail. Cells were lysed by passing the solution 4 times through an Emulsiflex High Pressure Homogenizer. The lysate was cleared by centrifugation (SS34, 30 min, 20’000 rpm, 4 °C), passed through a 0.23 μm pore size filter and loaded onto a 5 ml HisTrap HP column (GE Healthcare). The column was washed with four column volumes (CV) l&w buffer supplemented with 30 mM Imidazole and ten CV l&w buffer supplemented with 50 mM Imidazole. The protein was eluted in l&w buffer supplemented with 250 mM Imidazole in 1.5 ml fractions. Fractions containing the fusion protein His_6_Thioredoxin-Tev-^*Mtb*^Pup (^15^N or ^13^C^15^N) were pooled, supplemented with TEV protease and dialyzed against l&w buffer with 1 mM EDTA overnight at 4 °C using a 3 kDa molecular weight cutoff dialysis membrane. After 30 min heat denaturation at 90 °C, the solution was cleared by centrifugation, Thioredoxin and the TEV protease were removed by nickel affinity chromatography and the cleaved ^*Mtb*^Pup was further purified by size exclusion chromatography using a Superdex 75 pg column in 50 mM Tris, pH 7.6 (4 °C), 1 mM EDTA, and 150 mM NaCl for ^*Mtb*^PanB or 175 mM for ^*Mtb*^FabD.

### Identification of the pupylated lysine in ^*Mtb*^PanB and ^*Mtb*^FabD

Purified ^*Mtb*^PanB was pupylated as shown previously [5] and analyzed by SDS-PAGE. The gel band corresponding to the pupylated substrate protein was excised and subjected to tandem mass spectrometry analysis and the data were screened for peptides containing lysine with a GGE modification (Additional file 1: Figure S1a). Since no peptides carrying this modification were found, the two lysines K212 and K243 in undetected peptides remained candidates. K212 was mutated to alanine and the resulting ^*Mtb*^PanB variant was purified and tested in a standard pupylation reaction gel-shift assay (Fig. 1a for PanB-K212A).

The pupylation reaction performed with the ^*Mtb*^FabD-K122R-K181R mutant results in a heterogeneously pupylated ^*Mtb*^FabD as judged by the appearance of two protein bands on SDS-PAGE (Additional file 1: Figure S1b). To identify the additional pupylated lysine, all three bands (unpupylated and both pupylated bands) were excised and analyzed at the Functional Genomics Center Zurich using mass spectrometry analysis and the observed species were screened for peptides containing lysine with a GGE modification. In addition to the previously reported K173, we identified K35 to be modified with ^*Mtb*^Pup (modification + GGE, data not shown). This result was verified by additionally mutating K35 to alanine and testing the triple variant in the standard pupylation reaction (Additional file 1: Figure S1b).

### Substrate pupylation/NMR sample preparation

To ensure the ligation of all isotopically labeled ^*Mtb*^Pup (^15^N or ^13^C^15^N) to the respective target proteins, we performed the pupylation reaction with an excess of target protein. All pupylation reactions were carried out in reaction buffer containing 50 mM Tris-HCl, pH 8.0 (RT), 150 mM NaCl, 20 mM MgCl_2_ supplemented with 10 mM ATP. The reaction to generate ^15^N-^*Mtb*^Pup ~ ^*Mtb*^PanB or ^15^N-^*Mtb*^Pup ~ ^*Mtb*^FabD-3KR (Fig. 2) was performed in a total reaction volume of 2500 μl using 35 μM ^15^N-^*Mtb*^Pup, 40 μM ^*Mtb*^FabD-3KR or 40 μM ^*Mtb*^PanB in the presence of 2.5 μM of the Pup ligase ^*Cglu*^PafA-His_6_. The reaction mixture was incubated overnight at room temperature.

To generate the labeled ^13^C^15^N-^*Mtb*^Pup ~ ^*Mtb*^FabD-3KR sample, 40 μM of ^*Mtb*^FabD-3KR was incubated with 35 μM ^13^C^15^N-^*Mtb*^Pup in the presence of 10 mM ATP and 2.5 μM ^*Cglu*^PafA-His_6_ in 2050 μl total reaction volume for 13 h at 23 °C (Additional file 5: Figure S5). To generate labeled ^13^C^15^N-^*Mtb*^Pup ~ lysine, 50 mM L-lysine was incubated with 30 μM ^13^C^15^N-^*Mtb*^Pup in the presence of 10 mM ATP and 3 μM ^*Cglu*^PafA-His_6_ in 2350 μl total reaction volume for 300 min at 23 °C (Additional file 6: Figure S6).

Since the presence of the Pup ligase PafA during the NMR measurement would influence the conformation of Pup, we used a hexahistidine-tagged version of ^*Cglu*^PafA which was removed from the tagged substrates after the pupylation reaction (Fig. 2, Additional file 5: Figure S5 and Additional file 6: Figure S6) using Nickel affinity chromatography. After the removal of the Pup ligase ^*Cglu*^PafA, the reaction buffer was exchanged to a phosphate buffer containing 50 mM NaHPO_4_, 150 mM NaCl and 0.5 mM EDTA supplemented with Inhibitor Cocktail (Roche) using an Amicon spin column with 30 kDa molecular weight cutoff in the case of ^*Mtb*^Pup ~ ^*Mtb*^FabD or ^*Mtb*^Pup ~ ^*Mtb*^PanB and 3 kDa molecular weight cutoff in the case of ^*Mtb*^Pup ~ lysine. This step also ensured the removal of free unligated ^*Mtb*^Pup (in the case of ^*Mtb*^Pup ~ substrate production) or free lysine (in the case of ^*Mtb*^Pup ~ lysine production) from the sample.

The final concentration of the NMR samples was 300 μM for ^15^N-^*Mtb*^Pup, 185 μM for ^15^N-^*Mtb*^Pup ~ ^*Mtb*^FabD-3KR, 170 μM for ^15^N-^*Mtb*^Pup ~ ^*Mtb*^PanB, 124 μM for ^13^C^15^N-^*Mtb*^Pup ~ lysine and 98.5 μM for ^13^C^15^N-^*Mtb*^Pup ~ ^*Mtb*^FabD-3KR.

### NMR measurement

NMR experiments and data analysis was essentially performed as in [3]. The ^1^H chemical shifts were referenced to external DSS in the same buffer as the referenced sample. ^13^C and ^15^N shifts were referenced indirectly using published Ξ ratios [31].

NMR spectra were obtained using Bruker Avance 500, 600 and 700 MHz spectrometers equipped with three-channel cryoprobes. ^1^H^N^, ^13^Cα & ^15^N resonances of ^*Mtb*^Pup linked to ^*Mtb*^FabD-3KR where obtained with a ^13^C^15^N-^*Mtb*^Pup ~ ^*Mtb*^FabD-3KR sample recording standard triple resonance spectra at 10 °C. ^1^H^N^ and ^15^N resonance positions of ^*Mtb*^Pup linked to ^*Mtb*^PanB were obtained by transferring assignments from the positions of signals in ^15^N-^*Mtb*^Pup ~ lysine at a series of temperatures ranging from 10 °C to 25 °C. NMR data were processed using Topspin and analysed in CARA [32].

### Fluorescence anisotropy measurements

^*Cglu*^Pup-Q30C was labeled with fluorescein-5-maleimide as described before [13]. To generate ^*Cglu*^Pup-Q30C-Fluorescein ~ ^*Mtb*^FabD and ^*Cglu*^Pup-Q30C-Fluorescein ~ ^*Mtb*^PanB, 10 μM of ^*Cglu*^Pup-Q30C-Fluorescein was incubated with 20 μM ^*Mtb*^FabD or ^*Mtb*^PanB in the presence of 10 mM ATP and 2 μM ^*Cglu*^PafA-His_6_ in a total reaction volume of 200 μl for 5 h at room temperature. ^*Cglu*^PafA-His_6_ was removed as before using a Ni-NTA spin column and the flow-through was concentrated using an Amicon spin concentrator with molecular weight cutoff of 30 kDa. The concentration was determined by measuring absorbance at 492 nm and using an extinction coefficient of 83,000 M^−1^cm^−1^ (4 μM ^*Cglu*^Pup-Q30C-Fluorescein ~ ^*Mtb*^FabD and 5.5 μM ^*Cglu*^Pup-Q30C-Fluorescein ~ ^*Mtb*^PanB). All anisotropy measurements were performed on a Synergy BioTek plate reader using Corning® 96 Well Half Area Black Flat Bottom Polystyrene NBS™ Microplates (product code 3993). 12.5 nM of the ^*Cglu*^Pup-Q30C-Fluorescein labeled substrate or free ^*Cglu*^Pup was incubated with increasing concentrations of ^*Cglu*^PafA (0 to 30 μM) in 50 mM Tris, pH 8.04 (RT), 150 mM NaCl, 0.5 mM EDTA, 0.001% Tween-20. Determination of the affinity constant was performed as described in [13].

### Microscale thermophoresis (MST) affinity measurements

FabD-3KR pupylation was carried out as described for the Pup-substrate conjugates used in the NMR measurements.

^*Mtb*^Dop (7 μM) in 4 ml reaction volume (50 mM HEPES-NaOH pH 7.5 at 25 °C, 150 mM NaCl, 10% glycerol, 0.01% Tween-20) was fluorescently labeled by addition of 25 M equivalents of NHS-Fluorescein (Thermo Scientific, Prod# 46409, dissolved to 10 mg/ml in DMSO) and incubation at 25 °C for 3 h. The reaction mixture was concentrated to 500 μl and labeled ^*Mtb*^Dop was separated from excess Fluorescein by size exclusion chromatography on an Äkta purifier System (Amersham Biosciences) over a Superdex 200 gel filtration column (10/300) GL (GE healthcare). Collected fractions containing the labeled molecule were pooled and concentrated using Amicon tubes (Millipore, 30 kDa MWCO) to 7.08 μM ^*Mtb*^Dop-F with an average labeling ratio of 1.96 molecules of Fluorescein per enzyme.

Microscale Thermophoresis (MST) affinity measurements were conducted on a Monolith NT.115 device (Nanotemper), at a starting temperature of 19 °C with 50% blue LED power and 60% MST power in premium coated capillaries. Samples were prepared in 10 μl volume containing 30 nM ^*Mtb*^Dop-F and serial 1:1 dilutions of ^*Mtb*^Pup or ^*Mtb*^Pup ~ FabD-3KR from 25 μM to 15 nM in MST reaction buffer (50 mM HEPES-NaOH pH 7.5 at 25 °C, 150 mM NaCl, 1 mM MgCl_2_, 10% glycerol, 0.01% Tween-20). Measurements were performed in 3-7 technical replicates. The resulting curves were analyzed with the affinity analysis software (v2.0.2) provided by Nanotemper, using the built-in T-jump analysis protocol. The Kd fit was performed with the following equation, assuming a 1:1 binding stoichiometry:

Fraction bound = 1/(2 [^*Mtb*^Dop-F]) ([binder] + [^*Mtb*^Dop-F] + K_d_ - √(([binder] + [^*Mtb*^Dop-F] + K_d_)^2 – (4 [binder] [^*Mtb*^Dop])).

## Additional files

Additional file 1: Figure S1. Identification of the lysines of ^*Mtb*^PanB and ^*Mtb*^FabD modified with ^*Mtb*^Pup. **a** Amino acid sequence of ^*Mtb*^PanB. Those peptides identified from the ^*Mtb*^Pup ~ ^*Mtb*^PanB sample by mass spectrometry are indicated by *black bars* under the respective sequence stretches. All residues detected in fragments by the MS analysis are *colored red*. Potential pupylation target lysines are *highlighted in yellow*. **b** Pupylation reaction of 10 μM ^*Mtb*^FabD (wild type Strep-tagged compared with untagged double variant (K122R & K181R) or triple variant (K122R & K181R & K35R) in the presence of 10 μM ^*Mtb*^Pup, 10 mM ATP and 1 μM ^*Cglu*^PafA. The two bands corresponding to ^*Mtb*^FabD pupylated at K35 and ^*Mtb*^FabD pupylated at both K35 and K173 are marked inside the gel with *black arrows*. **c** Crystal structure of the ^*Mtb*^FabD monomer (pdb code 2QC3) shown in cartoon representation. All lysines are shown in space-filling representation (*green*: main target lysine, *orange*: secondary target lysines, *blue*: unmodified). (PNG 687 kb)

Additional file 2: Figure S2. Secondary shifts of ^*Mtb*^Pup ~ ^*Mtb*^FabD and ^*Mtb*^Pup ~ lysine. Chemical shifts of the native protein (^*Mtb*^Pup ~ Lys in *red*, ^*Mtb*^Pup ~ ^*Mtb*^FabD-3KR in *green*) minus shifts for ^*Mtb*^Pup ~ ^*Mtb*^PanB unfolded in Urea (data from [3]). The region with helical propensity in free ^*Mtb*^Pup is *indicated in grey*. (PNG 213 kb)

Additional file 3: Figure S3. [^15^N,^1^H]-HSQC spectra of ^15^N-labeled ^*Mtb*^Pup in free state or conjugated to ^*Mtb*^PanB. Superposition of spectra of ^15^N-^*Mtb*^Pup (*black*) and ^15^N-^*Mtb*^Pup ~ ^*Mtb*^PanB (*blue*), both measured at a temperature of 10 °C. (PNG 308 kb)

Additional file 4: Figure S4. ^*Mtb*^Pup covalently attached to a substrate remains accessible to ^*Mtb*^Dop. Binding of ^*Mtb*^Pup and ^*Mtb*^Pup ~ FabD-3KR to ^*Mtb*^Dop was analyzed using microscale thermophoresis (MST). Substrate (25 μM to 15 nM) was titrated to fluorescently labeled ^*Mtb*^Dop (30 nM). The resulting binding curves for ^*Mtb*^Pup (*red*) or ^*Mtb*^Pup ~ FabD-3KR (*green*) show a dissociation constant of 14.6 ± 3.3 nM and 157.4 ± 49.8 nM, respectively. (PNG 101 kb)

Additional file 5: Figure S5. ^13^C^15^N labeled ^*Mtb*^Pup ~ ^*Mtb*^FabD-3KR preparation and subsequent ^*Cglu*^PafA-His_6_ removal. Pupylation of 40 μM ^*Mtb*^FabD-3KR with 35 μM ^13^C^15^N-^*Mtb*^Pup in the presence of 10 mM ATP and 2.5 μM ^*Cglu*^PafA-His_6_ in 2050 μl total reaction volume and subsequent removal of the Pup ligase using a Ni-NTA spin-column. (PNG 244 kb)

Additional file 6: Figure S6. ^13^C^15^N labeled ^*Mtb*^Pup-Lysine preparation and ^*Cglu*^PafA removal. Pupylation of 50 mM L-Lysine with 30 μM ^13^C^15^N-^*Mtb*^Pup in the presence of 10 mM ATP and 3 μM ^*Cglu*^PafA-His_6_ in 2350 μl total reaction volume and subsequent removal of the Pup ligase using a Ni-NTA gravity flow column (FT: flow through, W1-W3: washes, E1, E2: elution with 250 mM Imidazole). (PNG 886 kb)

## Abbreviations

~: isopeptide linkage between Pup and its substrate
ATP: Adenosine triphosphate
Cglu: *Corynebacterium glutamicum*
Dop: Deamidase of Pup
DTT: Dithiothreitol
EDTA: Ethylene diamine tetra acetic acid
HSQC: Heteronuclear single quantum coherence
IPTG: Isopropyl β-D-1-thiogalactopyranoside
kDa: kilo Dalton
MS: Mass spectrometry
Mtb: *Mycobacterium tuberculosis*
NMR: Nuclear magnetic resonance
NTA: Nitrilotriacetic acid
OD: Optical density
PafA: Proteasome accessory factor A
PCR: Polymerase chain reaction
Pup: Prokaryotic ubiquitin-like protein
RT: Room temperature
SDS-PAGE: Sodium dodecyl sulfate polyacrylamide gel electrophoresis
TEV: Tobacco etch virus
UBD: Ubiquitin binding domain.
