# Supplementary figures and images for: Prokaryotic ubiquitin-like protein remains intrinsically disordered when covalently attached to proteasomal target proteins
Source: BMC Struct Biol. 2017 Feb 1;17:1. doi: 10.1186/s12900-017-0072-1 (PMC5286830; doi:10.1186/s12900-017-0072-1)

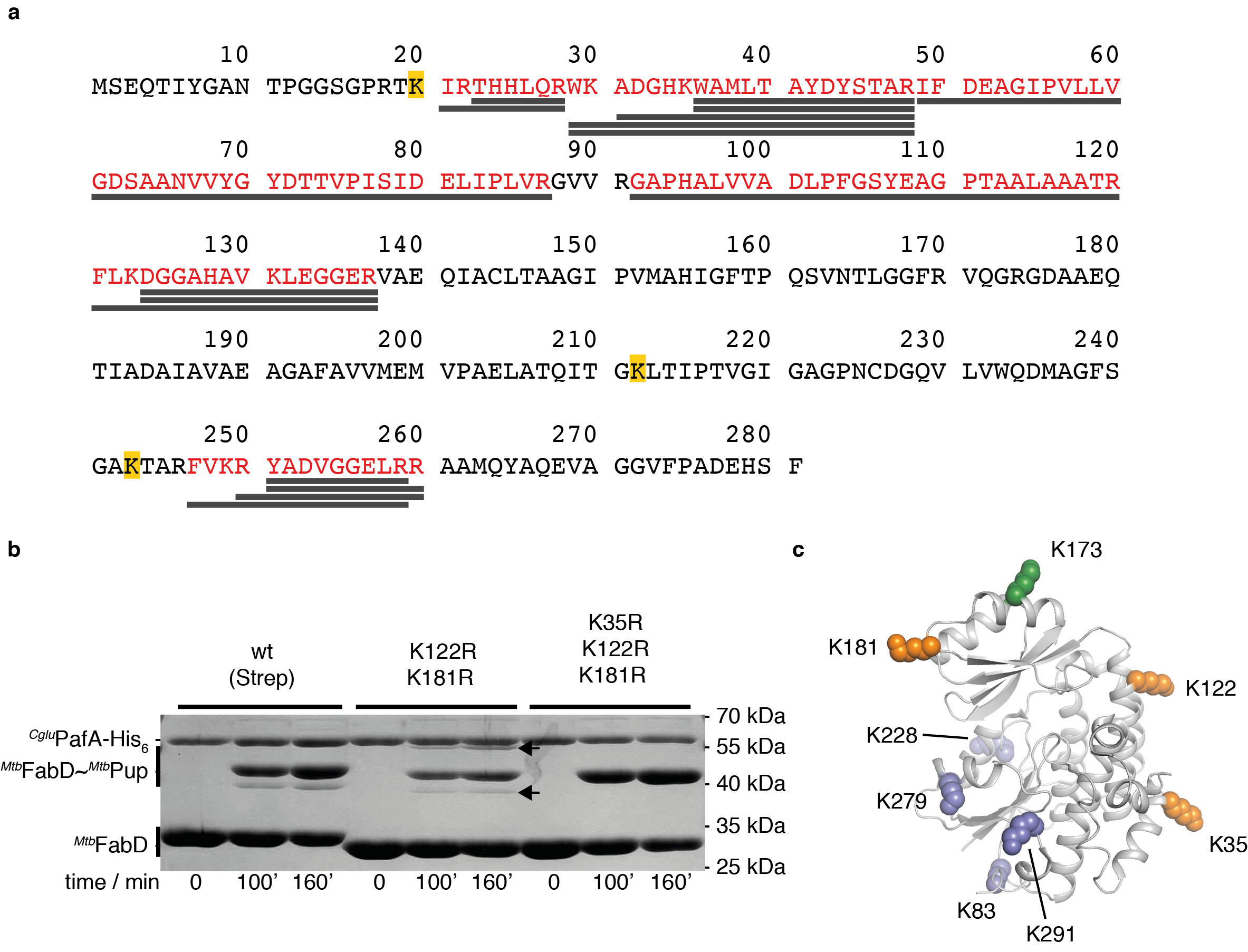

Supplement: Supplementary file 1 — Identification of the lysines of MtbPanB and MtbFabD modified with MtbPup. a Amino acid sequence of MtbPanB. Those peptides identified from the MtbPup ~ MtbPanB sample by mass spectrometry are indicated by black bars under the respective sequence stretches. All residues detected in fragments by the MS analysis are colored red. Potential pupylation target lysines are highlighted in yellow. b Pupylation reaction of 10 μM MtbFabD (wild type Strep-tagged compared with untagged double variant (K122R & K181R) or triple variant (K122R & K181R & K35R) in the presence of 10 μM MtbPup, 10 mM ATP and 1 μM CgluPafA. The two bands corresponding to MtbFabD pupylated at K35 and MtbFabD pupylated at both K35 and K173 are marked inside the gel with black arrows. c Crystal structure of the MtbFabD monomer (pdb code 2QC3) shown in cartoon representation. All lysines are shown in space-filling representation (green: main target lysine, orange: secondary target lysines, blue: unmodified). (PNG 687 kb) [file 12900_2017_72_MOESM1_ESM.png]

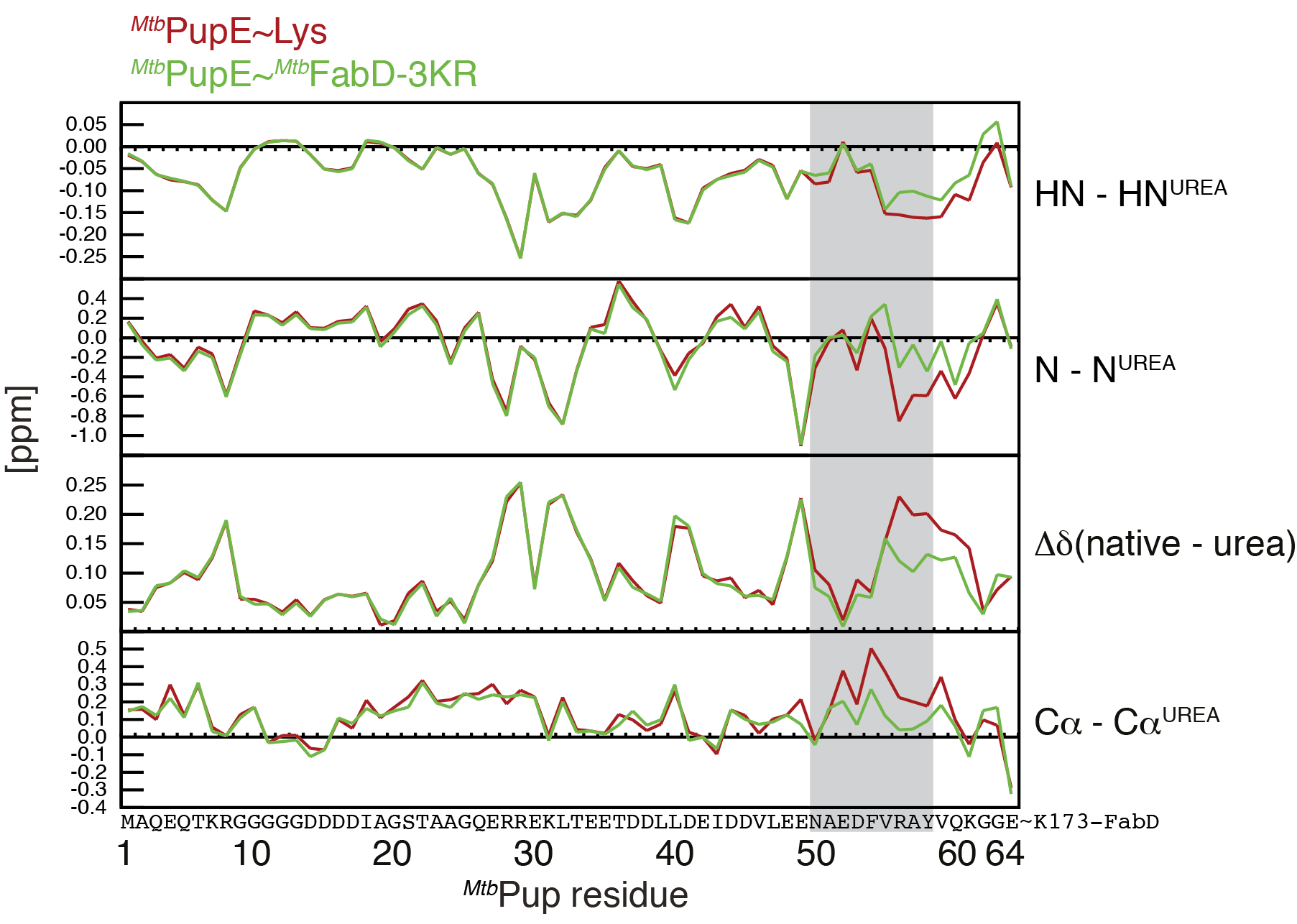

Supplement: Supplementary file 2 — Secondary shifts of MtbPup ~ MtbFabD and MtbPup ~ lysine. Chemical shifts of the native protein (MtbPup ~ Lys in red, MtbPup ~ MtbFabD-3KR in green) minus shifts for MtbPup ~ MtbPanB unfolded in Urea (data from [3]). The region with helical propensity in free MtbPup is indicated in grey. (PNG 213 kb) [file 12900_2017_72_MOESM2_ESM.png]

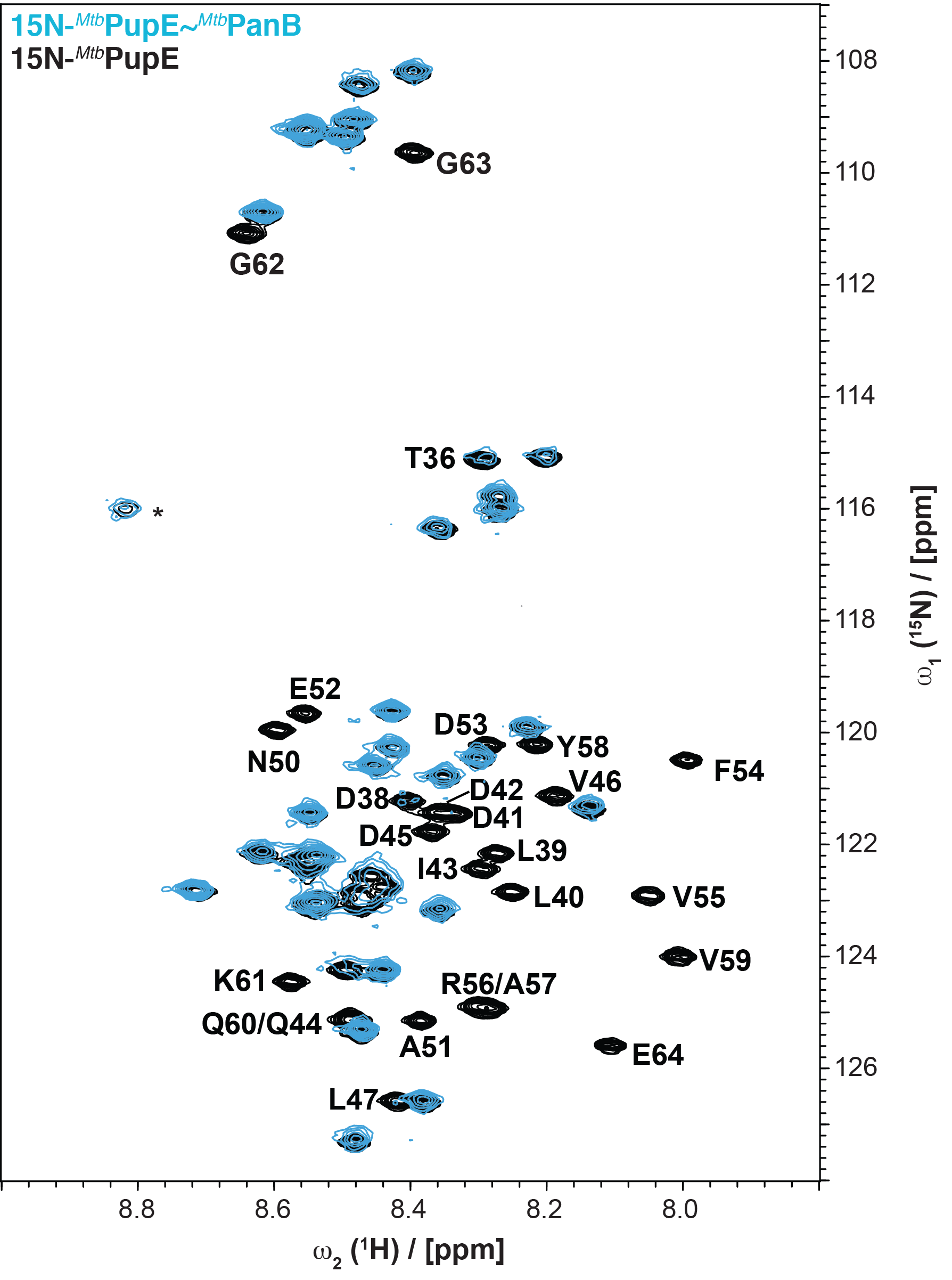

Supplement: Supplementary file 3 — [15N,1H]-HSQC spectra of 15N-labeled MtbPup in free state or conjugated to MtbPanB. Superposition of spectra of 15N-MtbPup (black) and 15N-MtbPup ~ MtbPanB (blue), both measured at a temperature of 10 °C. (PNG 308 kb) [file 12900_2017_72_MOESM3_ESM.png]

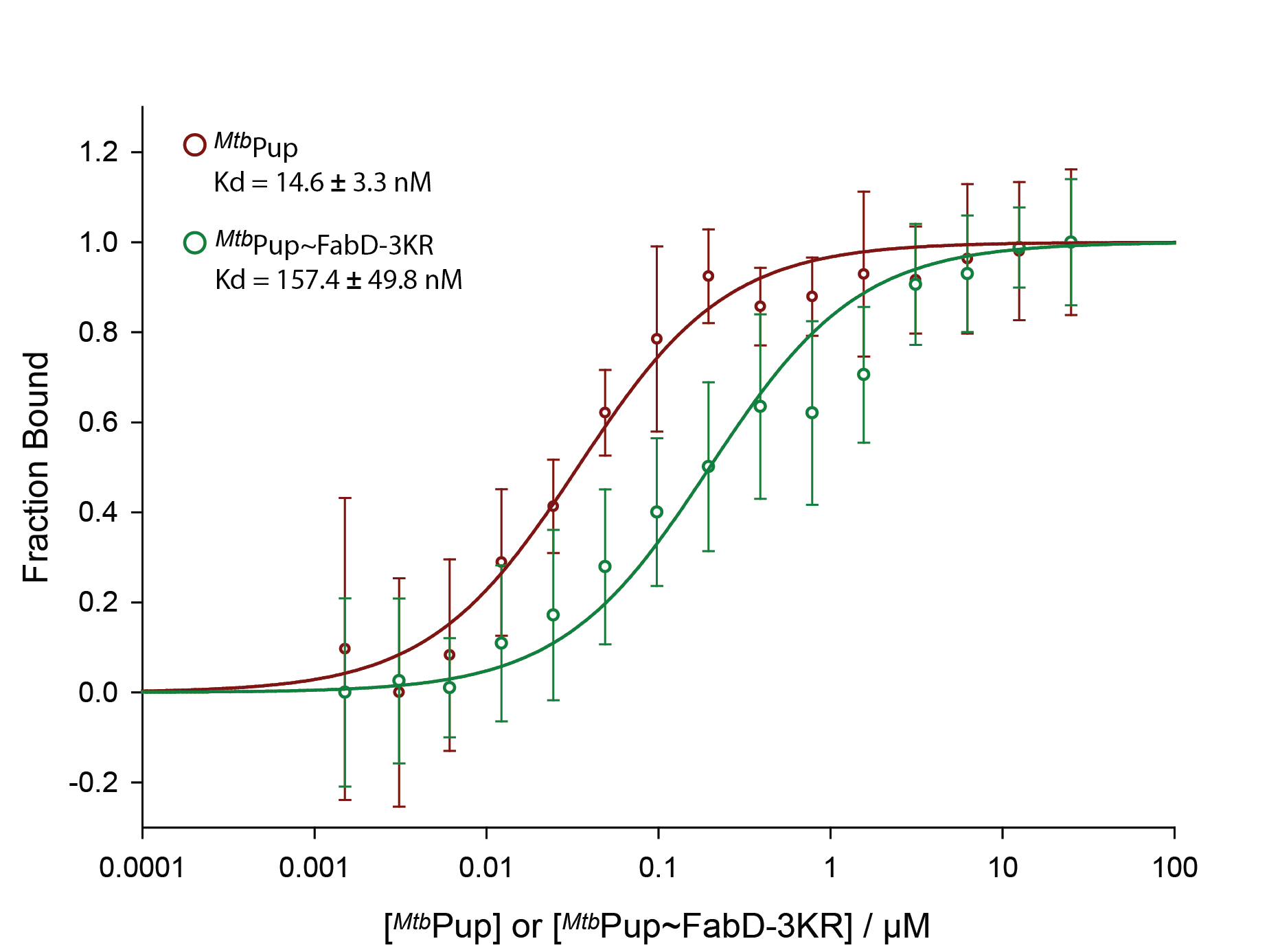

Supplement: Supplementary file 4 — MtbPup covalently attached to a substrate remains accessible to MtbDop. Binding of MtbPup and MtbPup ~ FabD-3KR to MtbDop was analyzed using microscale thermophoresis (MST). Substrate (25 μM to 15 nM) was titrated to fluorescently labeled MtbDop (30 nM). The resulting binding curves for MtbPup (red) or MtbPup ~ FabD-3KR (green) show a dissociation constant of 14.6 ± 3.3 nM and 157.4 ± 49.8 nM, respectively. (PNG 101 kb) [file 12900_2017_72_MOESM4_ESM.png]

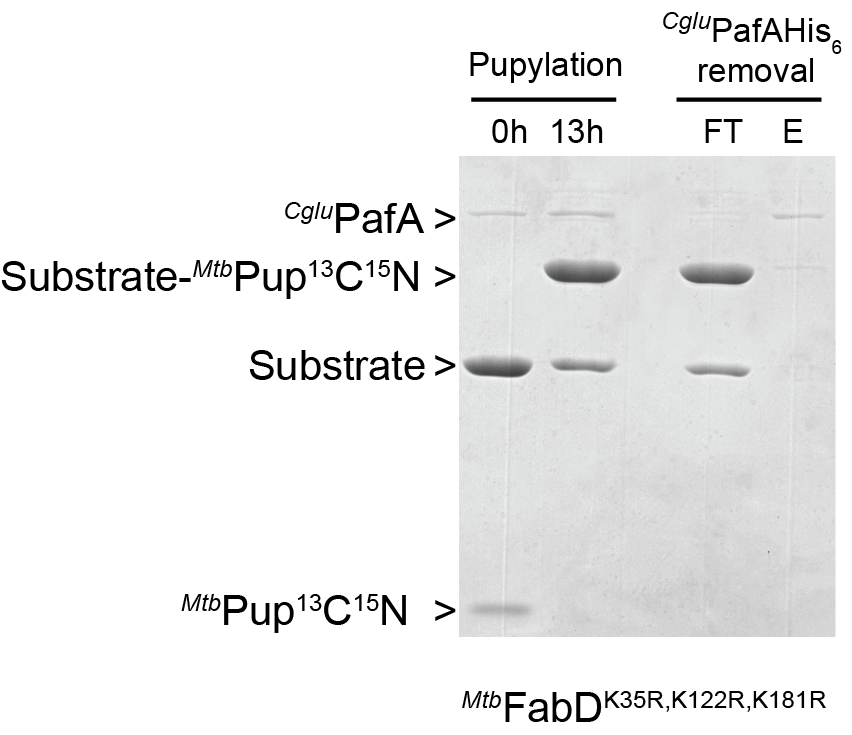

Supplement: Supplementary file 5 — 13C15N labeled MtbPup ~ MtbFabD-3KR preparation and subsequent CgluPafA-His6 removal. Pupylation of 40 μM MtbFabD-3KR with 35 μM 13C15N-MtbPup in the presence of 10 mM ATP and 2.5 μM CgluPafA-His6 in 2050 μl total reaction volume and subsequent removal of the Pup ligase using a Ni-NTA spin-column. (PNG 244 kb) [file 12900_2017_72_MOESM5_ESM.png]

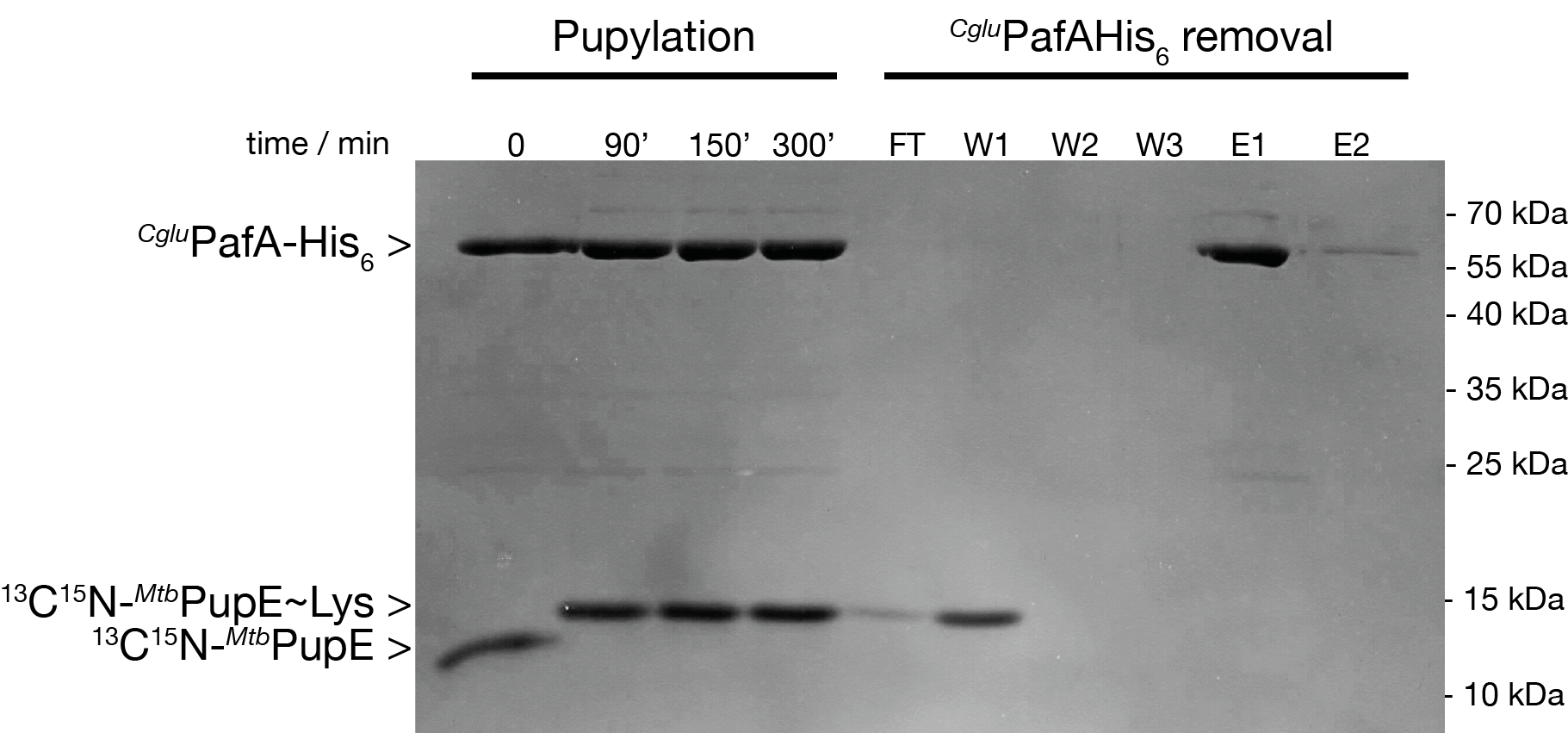

Supplement: Supplementary file 6 — 13C15N labeled MtbPup-Lysine preparation and CgluPafA removal. Pupylation of 50 mM L-Lysine with 30 μM 13C15N-MtbPup in the presence of 10 mM ATP and 3 μM CgluPafA-His6 in 2350 μl total reaction volume and subsequent removal of the Pup ligase using a Ni-NTA gravity flow column (FT: flow through, W1-W3: washes, E1, E2: elution with 250 mM Imidazole). (PNG 886 kb) [file 12900_2017_72_MOESM6_ESM.png]
